# Infection of Differentiated Porcine Airway Epithelial Cells by Influenza Virus: Differential Susceptibility to Infection by Porcine and Avian Viruses

**DOI:** 10.1371/journal.pone.0028429

**Published:** 2011-12-09

**Authors:** Darsaniya Punyadarsaniya, Chi-Hui Liang, Christine Winter, Henning Petersen, Silke Rautenschlein, Isabel Hennig-Pauka, Christel Schwegmann-Wessels, Chung-Yi Wu, Chi-Huey Wong, Georg Herrler

**Affiliations:** 1 Institute of Virology, University of Veterinary Medicine, Hannover, Germany; 2 Clinic for Poultry, University of Veterinary Medicine, Hannover, Germany; 3 Clinic for Swine and Small Ruminants, University of Veterinary Medicine, Hannover, Germany; 4 Genomics Research Center, Academia Sinica, Taipei, Taiwan; University of Hong Kong, Hong Kong

## Abstract

**Background:**

Swine are important hosts for influenza A viruses playing a crucial role in the epidemiology and interspecies transmission of these viruses. Respiratory epithelial cells are the primary target cells for influenza viruses.

**Methodology/Principal Findings:**

To analyze the infection of porcine airway epithelial cells by influenza viruses, we established precision-cut lung slices as a culture system for differentiated respiratory epithelial cells. Both ciliated and mucus-producing cells were found to be susceptible to infection by swine influenza A virus (H3N2 subtype) with high titers of infectious virus released into the supernatant already one day after infection. By comparison, growth of two avian influenza viruses (subtypes H9N2 and H7N7) was delayed by about 24 h. The two avian viruses differed both in the spectrum of susceptible cells and in the efficiency of replication. As the H9N2 virus grew to titers that were only tenfold lower than that of a porcine H3N2 virus this avian virus is an interesting candidate for interspecies transmission. Lectin staining indicated the presence of both α-2,3- and α-2,6-linked sialic acids on airway epithelial cells. However, their distribution did not correlate with pattern of virus infection indicating that staining by plant lectins is not a reliable indicator for the presence of cellular receptors for influenza viruses.

**Conclusions/Significance:**

Differentiated respiratory epithelial cells significantly differ in their susceptibility to infection by avian influenza viruses. We expect that the newly described precision-cut lung slices from the swine lung are an interesting culture system to analyze the infection of differentiated respiratory epithelial cells by different pathogens (viral, bacterial and parasitic ones) of swine.

## Introduction

Pigs are important hosts for influenza A viruses. Based on the surface antigens hemagglutinin and neuraminidase, influenza virus strains that are enzootic in swine populations worldwide are assigned to the subtypes H1N1, H3N2, or H1N2. Infection by other subtypes, e.g. H3N1, H4N6, H5N1 and H9N2 has been observed but they have not been maintained in pigs as independent lineages. Natural infections of pigs by influenza viruses from different hosts, e.g. by avian virus strains, have been reported [1]–[3]. It has been shown that infection of pigs with heterologous virus resulted in lower virus yields that failed to transmit infection to other pigs [4]. Though natural infections by avian influenza viruses were rarely able to establish a stable lineage in pigs, they may allow the introduction of new gene segments by genetic reassortment in host cells infected with two viruses. Influenza reassortants may not only provide the basis for the establishment of new lineages in pigs but also – after interspecies transmission – in new hosts. Therefore, pigs have been designated as mixing vessel for the combination of gene segments of viruses from different hosts [5].

Primary target cells for influenza viruses are cells of the respiratory epithelium. *In vitro* studies with differentiated respiratory epithelial cells are possible, e.g. by using air-liquid interface cultures or explant cultures. The former culture system has been used to analyze the infection by human influenza viruses [6], [7]. In the case of differentiated airway epithelial cells from pigs, infection studies with influenza viruses have been reported with explant cultures either from the trachea [8] or from different parts for the respiratory tract [9]. Here we report a new culture system for porcine differentiated respiratory epithelial cells, precision-cut lung slices (PCLS). This culture system has been used for various scientific fields, but rarely for infection studies [10], [11]. Interesting features of PCLS are that (i) they can be obtained in large numbers, (ii) differentiated epithelial cells are maintained in their original setting, and (iii) they are viable for more than a week. Here we used this culture system to compare the infection of respiratory epithelial cells by a swine and two avian influenza A viruses. Interestingly, porcine airway epithelial cells are much more susceptible to an avian virus of the H9N2 subtype than to an H7N7 virus.

## Results

### Precision-cut lung slices (PCLS), a model system for differentiated porcine respiratory epithelial cells

Differentiated cells of the respiratory epithelium are the target cells for influenza viruses. In order to analyze the infection by porcine influenza virus, we established a culture system for differentiated respiratory epithelial cells from the porcine lung. For this purpose we prepared precision-cut lung slices from the lung of three months old animals. For infection studies, so far only PCLS from bovine, murine and avian lungs have been used. In order to determine whether PCLS from the porcine lung are a suitable culture system for infection studies, the vitality of the epithelial cells was determined.

A characteristic feature of the bronchial epithelium is the presence of ciliated cells. In slices presenting the circular epithelium lining a bronchus or bronchiolus, the ciliary activity was monitored by light microscopy at daily intervals. The ciliary activity was found to be fully maintained up to nine days post preparation provided that the medium is changed at daily intervals (data not shown). From day ten on, the ciliary activity was estimated to be decreased to 90% indicating that small areas comprising about 10% of the epithelial lining did not show movement of the cilia. This level of ciliary activity was maintained for additional five days at least (data not shown).

**Figure 1:**
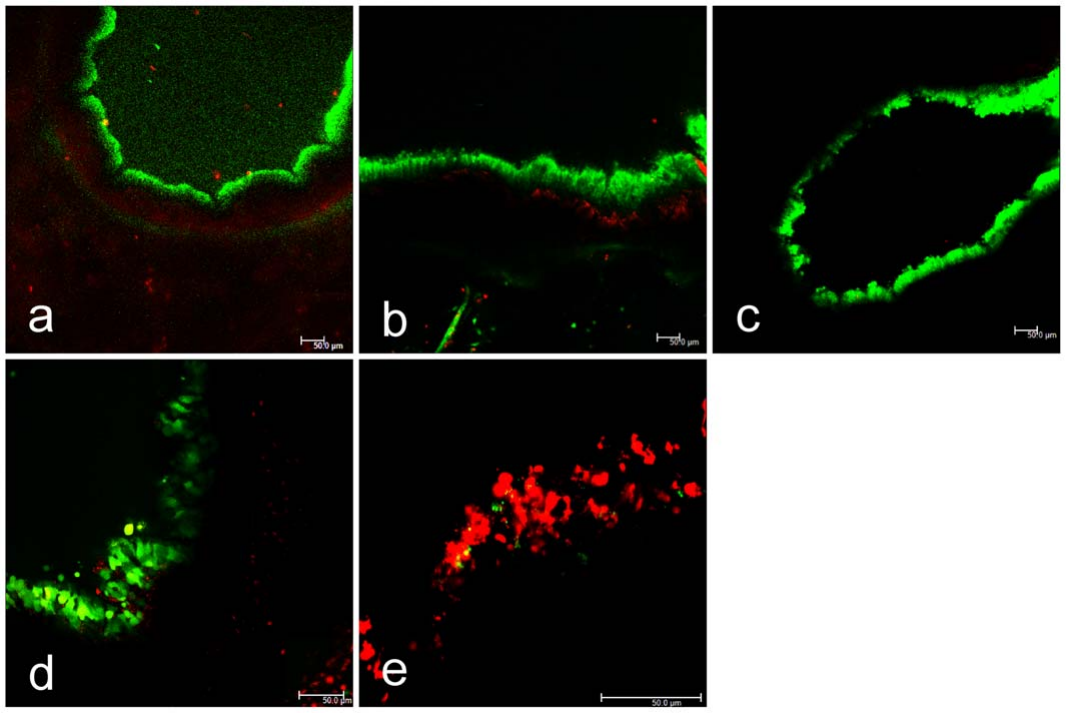
Vitality of PCLS evaluated by live (green)/dead (red) staining. Slices were stained with a commercial kit at day 1, 3 and 7 after preparation (upper panels, a–c). For comparison, in the lower panels the live/dead staining is shown for two slices that either had retained full ciliary activity (100%) (d), or had completely lost ciliary activity (0%) (e). The scale bar indicates 50 µm.

The vitality of the PCLS was also analyzed by subjecting the cells to a live/dead viability/cytotoxicity assay. As shown in Fig. 1 (upper panel), the epithelial lining of a bronchus consisted mainly of viable cells (green) when analyzed at 1, 3 or 7 days post-preparation (Fig. 1a–c). The distribution of the red stain indicates that only very few cells of the epithelial lining were dead. To relate the ciliary activity to the live/dead staining, in the lower panel two slices are shown with 100% (Fig. 1d) or 0% (Fig. 1e) ciliary activity. In the latter case, the majority of cells are red, i.e. dead.

**Figure 2:**
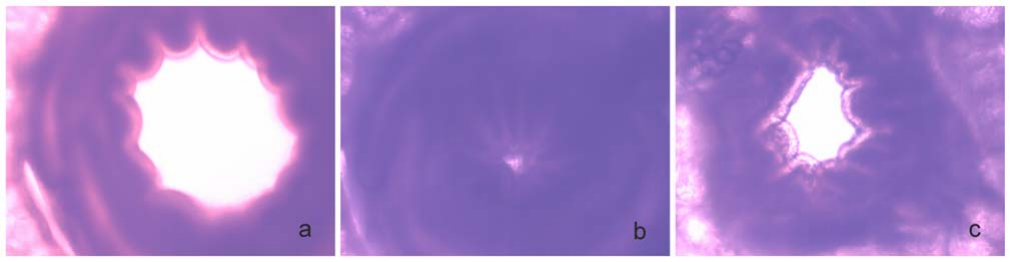
Vitality of PCLS evaluated by bronchoconstriction. To induce bronchoconstriction, the untreated slice (a) was incubated with 10^−4^ M methacholine (b). Removal of the drug resulted in a reverse effect (c).

As a further criterion for the vitality of PCLS we analyzed whether bronchoconstriction can be induced in a reversible manner. As shown in Fig. 2, addition of methacholine at a concentration of 10^−4^ M resulted within few minutes in bronchoconstriction as indicated by the complete closure of the bronchus (compare Fig. 2, a and b). When the drug was removed from the sample, the bronchus opened again in a process that took about one hour. Bronchoconstriction was observed with PCLS that were analyzed 1, 3 or 7 days after preparation (not shown).

### Expression of sialic acid

**Figure 3:**
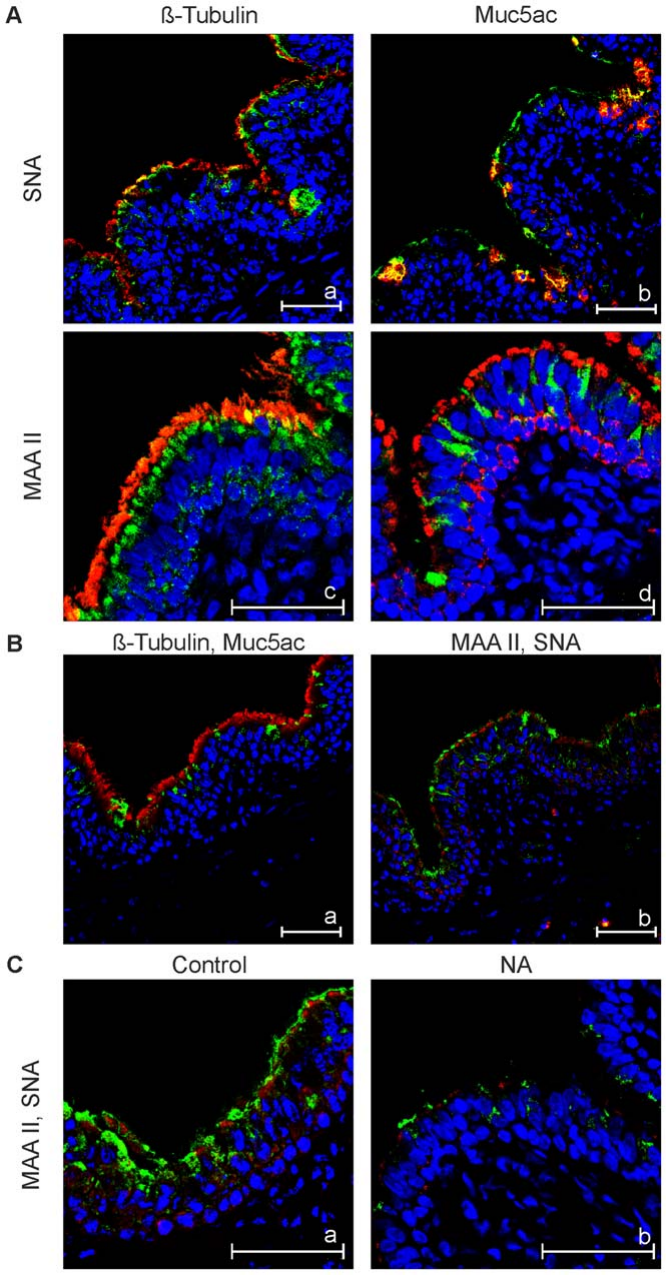
Expression of sialic acid in swine PCLS cells. Sialic acids were detected by lectin staining: MAA (*Maackia amurensis agglutinin*) for α2,3-linked sialic acids and SNA (*Sambucus nigra agglutinin*) for α2,6-linked sialic acids. Ciliated cells were stained using an anti-β-tubulin antibody and mucus-producing cells were stained using an anti muc5ac antibody. In panels A, lectin-staining is compared with staining of ciliated (red) or mucus-producing cells (red in Ab, green in Ad); SNA staining is shown in green (Aa and Ab), MAA staining is shown in green (Ac) or red (Ad). In Ba, co-staining of ciliated (red) and mucus-producing cells (green) is shown; Bb shows co-staining with MAA (red) and SNA (green). In C samples were co-stained SNA (green) and MAA (red); cryosections were derived from PCLS before (Ca) or after neuraminidase treatment (Cb).

As sialic acids are crucial receptor determinants in the entry process of influenza viruses, we analyzed PCLS for the expression of sialic acids. Cryosections from PCLS were subjected to a staining procedure based on the sialic acid-specific lectins *Sambucus nigra agglutinin* (SNA) and *Maackia amurensis agglutinin* (MAA) type ΙΙ. The former lectin recognizes α-2,6-linked sialic acids, the latter α-2,3-linked sialic acids. To differentiate between cell types, slices were stained for the presence of ß-tubulin to detect ciliated cells and for the presence of mucin to detect mucus-producing cells. The distribution of ciliated (red) and mucus-producing cells (green) is shown in Fig. 3Ba. As the anti-tubulin antibody primarily reacts with cilia, the red stain is concentrated on the cell surface of the ciliated epithelium. By contrast, the Muc5ac antibody stains mucus droplets and the cells containing mucus. The distribution of cells stained by the two lectins is shown in Fig. 3Bb. Though the intensity of the SNA staining (green) may suggest that α-2,6-linked sialic acids are more prominent on the bronchial epithelium than are α-2,3-linked sialic acids, the MAA staining (red) was also readily detectable. The staining pattern observed with the two lectins was different. Whereas MAA binding is primarily detected at the luminal surface, SNA staining was also observed at lateral sites. This difference was also evident when the staining with either of the lectins was performed together with the staining of a marker for ciliated or mucus-producing cells. As shown in Fig. 3A, the green staining pattern obtained with SNA went in parallel with both the anti-tubulin (Fig. 3Aa) and anti-Muc5ac staining (3Ab), indicating that both ciliated and mucus-producing cells express α-2,6-linked sialic acids. By contrast, the MAA staining pattern only resembled that of ciliated cells (Fig. 3Ac, red for tubulin, green for MAA), but not that of mucus-producing cells (Fig. 3Ad, red for MAA, green for Muc5ac) suggesting that α-2,3-linked sialic acids are mainly found on ciliated cells. Pretreatment of PCLS by neuraminidase from *Clostridium perfringens* abolished lectin-specific staining (compare Figs. 3Ca and 3Cb) indicating that binding of both MAAII and SNA was sialic acid-specific.

### Virus infection of precision-cut lung slices (PCLS)

**Figure 4:**
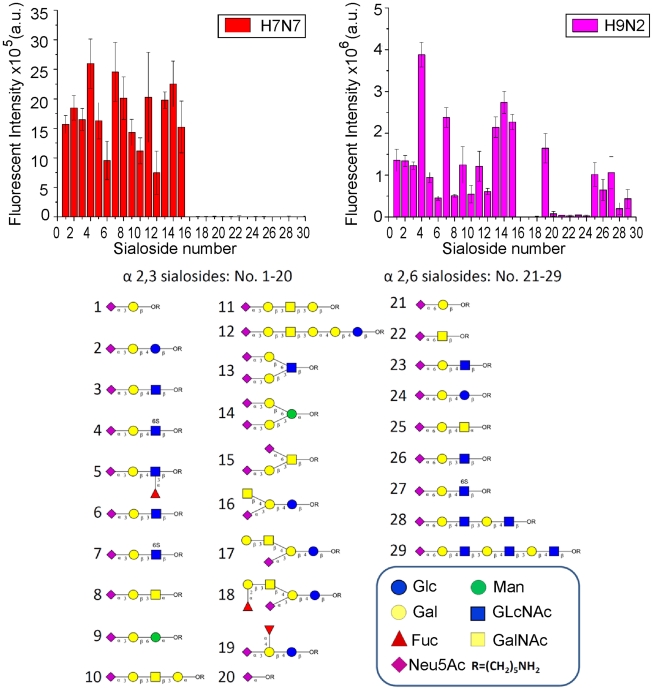
Glycan array analysis. Avian influenza virus (H9N2 and H7N7 subtypes) were subjected to glycan array analysis.

For our comparative analysis of the infection of differentiated porcine respiratory epithelial cells, we chose a porcine influenza virus of the H3N2 subtype (strain A/sw/Bissendorf/IDT1864/2003) and two avian viruses of the H7N7 (strain A/duck/Potsdam/15/80) and H9N2 (strain A/chicken/Saudi Arabia/CP7/98) subtype, respectively. Viruses of the former subtype have been shown to preferentially recognize α-2,6-linked sialic acids [12]. Most avian viruses have a preference for α-2,3-linked sialic acids [13]. Avian influenza viruses of the H9N2 subtype have been reported to bind to both α-2,3- and to α-2,6-linked sialic acids. Strains containing a leucine rather than a glutamine at position 226 of the hemagglutinin, even have a preference for α-2,6-linked sialic acids [14]. To characterize the receptor binding activity of the avian strains used in this study, a glycan array analysis was performed. As shown in Fig. 4, the H7N7 virus exclusively recognized glycans containing α-2,3-linked sialic acids. By contrast, the H9N2 virus bound to both α-2,3- and α-2,6-linked sialic acids. In general, the H7N7 virus recognized α-2,3-linked sialic acids with higher affinity. Among 20 sialosides containing α-2,3-linked sialic acids, there was only one (sialoside 19) that was recognized by the H9N2 but not by the H7N7 virus. The finding that the H9N2 virus recognized α-2,6-linked sialic acids with lower affinity compared to α-2,3-linked sialic acids is consistent with the fact that this virus contained a glutamine at position 226 of the hemagglutinin.

**Figure 5:**
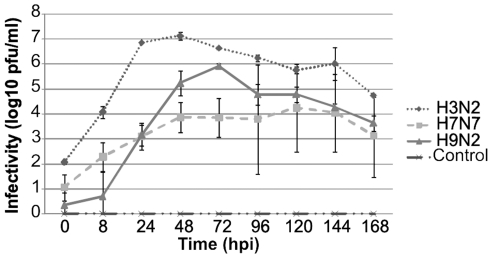
Infection of PCLS by porcine and avian influenza viruses evaluated by titration of infectious virus. PCLS were mock-infected or infected by porcine H3N2, avian H9N2, or avian H7N7 virus. Up to 7 days post infection, infectious virus released into the supernatants of PCLS was titrated at daily intervals by plaque assays.

To analyze the infection of well-differentiated respiratory epithelial cells by swine influenza virus, PCLS were infected by strain A/sw/Bissendorf/IDT1864/2003 (H3N2). The efficiency of infection was determined by titration the infectious virus in the supernatant at different time points after infection. As shown in Fig. 5, for the swine influenza virus an infectivity of about 10^7^ pfu/ml was determined at 24 h.p.i. which was only slightly increased by 48 h.p.i. For comparison, the two low-pathogenic avian influenza virus strains mentioned above were included in our analysis, A/chicken/Saudi Arabia/CP7/98 (H9N2) and A/duck/Potsdam/15/80 (H7N7). The growth of the two avian viruses differed from that of the swine virus both in the maximum titer and in the time course of virus release into the supernatant. The H9N2 virus reached a titer of about 10^5^ pfu/ml at 48 h.p.i. which increased to 10^6^ pfu/ml by 72 h.p.i. Thus, compared to the swine influenza virus this avian virus grew to a 10-fold lower titer in a time period that was prolonged for about 24 h. The H7N7 virus resembled the H9N2 in the time course but reached infectivity values of only 10^4^ pfu/ml.

### Effect of influenza virus infection on the ciliary activity

**Figure 6:**
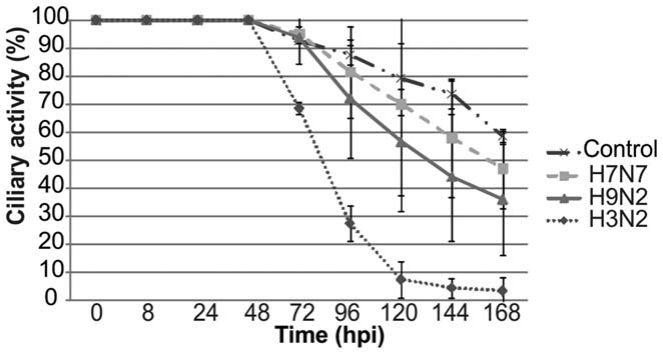
Infection of swine PCLS by porcine and avian influenza viruses evaluated by ciliary activity. PCLS were mock-infected or infected by porcine H3N2, avian H9N2, or avian H7N7 virus. Up to seven days post infection, PCLS were analyzed for ciliary activity at daily intervals.

The infection of PCLS by influenza virus was also analysed for its effect on the viability of the epithelium. For this purpose, the ciliary activity was determined and found to be unaffected by 48 h.p.i. (Fig. 6). After the second day, ciliary activity was decreased in all infected samples. However, while almost complete ciliostasis was observed at day five after infection with the swine influenza virus, more than 50% of the epithelial surface analyzed retained the ciliary activity in PCLS until day five after infection with either of the two avian virus strains. Thus, the lower efficiency of infection of the avian viruses revealed in Fig. 5 was reflected by a less pronounced ciliostatic effect. It should be noted that a decrease of the ciliary activity was observed also in the control sample. This effect is explained by the fact that the medium is not changed after infection.

### Immunostaining of swine PCLS infected by swine and avian influenza virus

**Figure 7:**
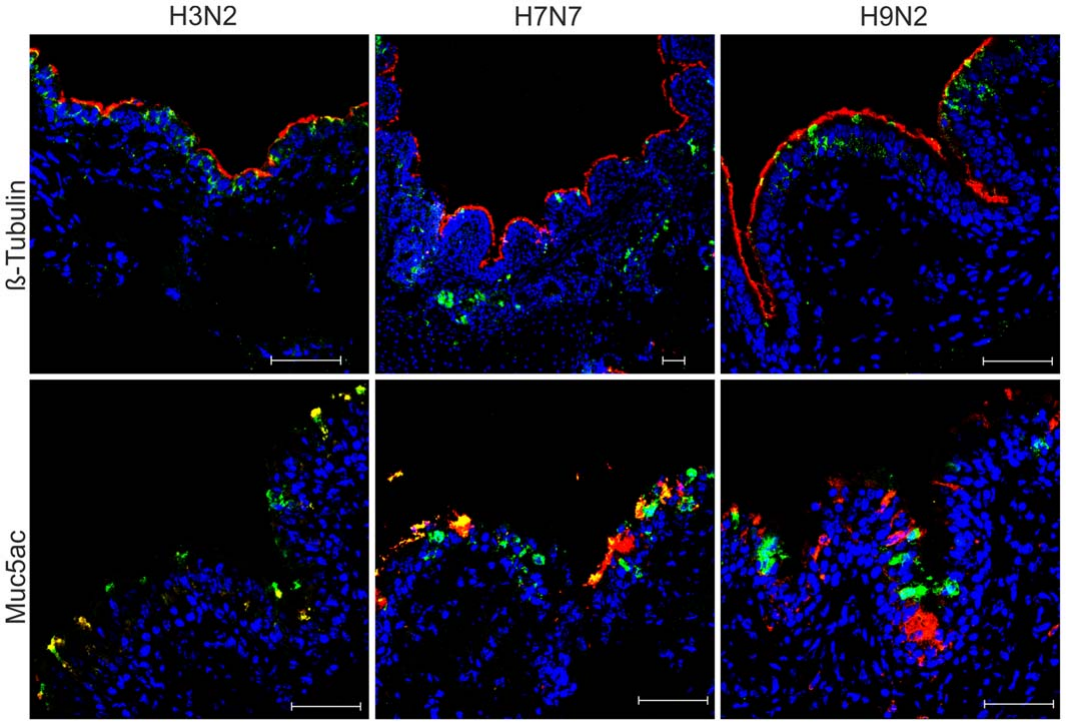
Infection of swine PCLS by influenza viruses characterized by immunostaining. PCLS were infected by either porcine H3N2, avian H9N2, or avian H7N7 virus. Cryosections were prepared at 24 h.p.i. and used for detection of infected cells, ciliated cells, and mucus-producing cells. Infected cells were stained with an anti-nucleoprotein antibody (green); ciliated cells were stained using an anti-β-tubulin antibody (red) and mucus-producing cells were stained using an Muc5Ac antibody (red).

To detect infected cells, cryosections of swine PCLS were stained for the presence of viral antigen using an antibody directed against the nucleoprotein. For comparison, the cryosections were stained in parallel for the presence of ciliated and mucus-producing cells (Fig. 7, in red). More infected cells were detected in PCLS inoculated with swine influenza virus (left panels) when compared to the samples infected by the two avian viruses, H7N7 (middle panels) and H9N2 (right panels). Whereas infections by swine influenza virus and the H9N2 virus, respectively, were limited to the epithelial cells lining the bronchus, H7N7-infected cells were also detected in submucosal cell layers. Co-staining of cryosections for the presence of viral antigen and tubulin revealed that all three viruses were able to infect ciliated cells (Fig. 7, upper panels). Co-staining of PCLS for the presence of infected and mucus-producing cells (Fig. 7, lower panels) showed clear evidence that mucus-producing cells were infected by swine influenza virus (left panel) and the H7N7 avian virus (middle panel) whereas mucus-producing cells infected by H9N2 virus were detected only occasionally.

## Discussion

PCLS have been used for more than ten years to address pharmacological, toxicological, or physiological questions related to airways of different species [15], [16]. Reports about infection studies with PCLS are rare and restricted to the murine, bovine, and avian lungs [10], [11], [17], [18]. In this study, PCLS prepared from porcine lungs were shown to be a valuable culture system for porcine differentiated respiratory epithelial cells. The vitality markers used indicate that the ciliated epithelium remains intact for more than one week when the medium is changed daily. For comparison, conventional porcine airway explant cultures have been reported to be viable for four days [9]. In the latter analysis, explants were obtained by manual cutting which may not preserve the tissue as well as in the case of precision-cut lung slices generated by the tissue slicer. Furthermore, prior to preparation of PCLS, airways are stabilized by filling with low-melting agarose. By, contrast for the conventional explants mentioned above, stabilization of the airways was applied to lung explants but not to bronchiolar explants, and for lung explants, the method involved two agarose embedding steps rather than one as in our case. These methodical differences may explain that epithelial cells in PCLS are better preserved than in conventional explant cultures. Further advantages of PCLS are that the ciliary activity and bronchoconstriction can be used as indicators of the intactness of the epithelium and thus for the cytopathic effect of a virus infection.

The appropriateness of PCLS for infection studies with influenza viruses is shown by the high sensitivity to infection. Both porcine and avian virus strains were applied at a concentration of 10^4^ pfu/ml. In a recent study with conventional explant cultures, 10^6^ TCID_50_/ml were used to obtain comparable virus titers in the supernatant [9]. A difference between infection by the porcine and the avian viruses was evident not only in the amount of virus released into the supernatant but also in the time course of virus release. In comparison to the porcine influenza virus, growth of the two avian virus strains was delayed by about 24 h; despite the different time course of virus production, the ciliary activity was maintained up to 48 h.p.i. with all three viruses. Ciliostasis appeared to be dependent on the amount of virus generated during infection. In PCLS infected with the swine influenza virus, ciliostasis started at day three and was almost complete by day five after infection. In contrast, only partial ciliostasis was observed in PCLS infected with the avian viruses; a substantial portion of the epithelium in the microscopic field had retained ciliary activity even at day seven post-infection.

Immunofluorescence analysis revealed that porcine alveolar epithelial cells can be infected by influenza viruses (data not shown) though at lower frequency than cells of the bronchiolar section of the respiratory tract. However, as we were not able to differentiate between porcine type I and type II pneumocytes, our analysis focussed on the bronchiolar epithelium. We found that all three viruses were able to infect ciliated epithelial cells. The porcine influenza virus also infected mucus-producing cells. This tropism of the porcine H3N2 virus for both ciliated and non-ciliated cells appears to be different from the respective human viruses which have been reported to show a preference for nonciliated human airway cells [6]. However, other authors presented data showing infection of both ciliated and nonciliated cells by human influenza viruses [7]. Interestingly, the two avian viruses differed in the spectrum of infected cells. While the H9N2 virus mainly infected ciliated cells, the H7N7 virus also infected mucus-producing cells and furthermore, cells in the submucosal area. The spectrum of susceptible cells does not reflect the efficiency of virus replication. The amount of infectious H9N2 virus released into the supernatant was only about tenfold lower than that determined for the infection by the porcine virus. On the other hand, with the H7N7 virus which had the broadest spectrum of susceptible cells in the bronchial epithelium, maximum virus titers were 100-fold lower when compared to the H9N2 virus. There is no straightforward explanation for the differences observed between the two avian viruses. Sialic acids present on the cell surface are crucial for the entry of influenza viruses into target cells. However, with only two plant lectins available for differentiation among sialic acids, it is not possible to draw conclusions about the presence of appropriate receptors for the entry of influenza viruses. This statement is even more valid when it is taken into account that reports about the presence of sialic acids in the respiratory tract of pigs appear to be controversial. While there is agreement that binding sites for the lectin SNA (α2,6-linked sialic acids) are abundantly present, we (this report) and others [19], [20] found that staining by the lectin MAAII (specific for α2,3-linked sialic acids) is also readily detectable. By contrast, in other reports authors found some staining by MAAII in the lower airways but very little staining in the trachea and bronchi [9], [21] Anyway, the lectins used are expected to stain the most abundant sialoglycoconjugates, i.e the mucins generated by mucus-producing cells and the glycocalix on the surface of the ciliated epithelium. Thus, the results of the lectin staining indicate that mucins present in PCLS contain α2,6-linked sialic acids and that both α2,3- and α2,6-linked sialic acids are present in the glycocalix on the surface of the ciliated epithelium. While these cellular components may provide primary attachment sites, it is not known which sialoglycoconjugates are able to mediate endocytotic uptake of influenza viruses. Those macromolecules may be present in much lower amounts and it is not known whether they can be stained by any of the plant lectins. These considerations have to be kept in mind to understand the difference between the two avian viruses in the spectrum of susceptible cells. As avian influenza viruses in general have a preference for α2,3-linked sialic acids as receptor determinants it may appear surprising that the H7N7 virus is able to infect mucus-producing cells because they were stained only with SNA and not with MAA. However, the presence of α2,6-linked sialic acids on mucins does not exclude that the sialoglycoconjugates required on the cell surface for the entry process of influenza viruses contain α2,3-linked sialic acids. Furthermore, mucins may also be a barrier for virus entry that has to be inactivated by appropriate viral neuraminidases [22]. Such considerations may provide explanations for the different ability of H7N7 and the H9N2 virus to infect mucus-producing cells, if infection by the latter virus is prevented at the stage of virus entry. However, it is also possible that post-entry steps affect the differential replication efficiency of the H7N7 and the H9N2 virus. In this context it is also interesting that the avian H7N7 virus was able to infect some submucosal cells even though these cells showed a much lower reactivity in the MAA staining than the ciliated epithelium. This finding is consistent with the considerations discussed above that staining by plant lectins is not a reliable indicator for the presence or absence of receptors for influenza viruses. This statement is in agreement with a report that *ex vivo* cultures of human nasopharyngeal, adenoid and tonsillar tissues can be infected with H5N1 viruses despite the difficulty of detecting α2,3-linked sialic acids by MAA staining [23].

The high titers of the H9N2 virus generated in porcine airway epithelial cells appear to be a unique feature of this avian virus. In a recent study with explant cultures, van Poucke and coworkers [9] analyzed six different avian virus strains comprising the subtypes H1N1, H3N8, H5N1, H4N6, H5N2, and H7N1. These authors reported that the virus titers in the bronchial epithelium were in the range between 1×10^3^–1×10^5^TCID_50_/ml, i.e not higher than we determined for the H7N7 virus. Thus, replication of the H9N2 virus may be less restricted in the porcine airways than that of other avian viruses. Therefore, for this virus it may be easier than for other avian viruses to cross the barrier to mammalian species which is consistent with the finding that H9N2 infections of man and swine have occurred previously [3], [24]. Future work should address the adaptation process of the H9N2 virus in porcine PCLS. It will be of special interest to know how many passages are required for this avian virus to reach titers as high as that determined for the swine influenza virus and whether the adaptation is evident in a shorter replication time or in a higher viral yield or in both parameters.

## Materials and Methods

### Ethics statement

Pigs used for these experiments were kept in the Clinic for Swine and Small Ruminants for demonstration and studentsì veterinary training (approval number 33.9-42502-05-09A627). All studies were carried out in strict accordance with the recommendations of the European Convention for the Protection of Vertebrate Animals used for Experimental and Other Scientific Purposes (European Treaty Series, nos. 123 [http://conventions.coe.int/Treaty/en/Treaties/Html/123.htm] and 170 [http://conventions.coe.int/Treaty/en/Treaties/Html/170.htm]. The protocol was approved by the national permitting authorities (animal welfare officer of the University of Veterinary Medicine, Lower Saxony State Office for Consumer Protection and Food Safety). All measures were in accordance with the requirements of the national animal welfare law. Killing and tissue sampling were performed under sodium pentobarbital anesthesia, and all efforts were made to minimize suffering.

### Precision-cut lung slices (PCLS)

PCLS were prepared from lungs of three months old crossbred pigs originated from conventional farms and housed in the Clinics for Swine and Small Ruminants and Forensic Medicine at the University of Veterinary Medicine, Hannover. Pigs showed no clinical symptoms of respiratory or systemic disease. Immediately after euthanasia with pentobarbital, lungs were carefully removed and the cranial, middle, and intermediate lobes were filled with 37°C warm low-melting agarose (agarose LM GQT; GERBU, Gaiberg, Germany) followed by solidification on ice. Tissue was stamped out as cylindrical portions (8-mm tissue coring tool) and approx. 250 µm thick slices were prepared by using the Krumdieck tissue slicer (TSE systems, model MD4000-01) with a cycle speed of 60 slices/min. PCLS were incubated in 1 ml of RPMI 1640 medium (Invitrogen/Gibco, Germany) containing antibiotics and antimycotics (Amphtericin B, Clotrimazole, Enrofloxacin, Kanamycin, Penicillin/Streptormycin) per slice in a 24-well plate at 37°C and 5% CO_2_. The medium was changed every hour during the first four hours and once after 24 hours, before slices were used for infection.

The viability was analyzed by observing the ciliary activity under the light microscope (Zeiss Axiovert 35) equipped with an ORCA C4742-80 digital camera (Hamamatsu) and SIMPLE-PCI analysis software (Compix Imaging Systems). In selected samples, the slices were analyzed for bronchoconstriction by addition of 10^−4^ M methacholine (acetyl-ß-methylcholine chloride, Sigma Aldrich). The integrity of the cells was also determined by applying a Live/Dead viability/cytotoxicity assay kit (Fluo Probes, FP-BE4710). For this purpose, the slices were washed with phosphate-buffered saline (PBS) and incubated with Calcein AM (1 µM) and ethidium bromide (EthD-1; 2 µM) for 30 minutes. After incubation, slices were washed with PBS, embedded in Mowiol resin and analyzed using a Leica TCS SP5 AOBS confocal laser scanning microscope.

### Madin-Darby canine kidney (MDCK) cells

MDCKII cells were maintained in Eagle's minimal essential medium (EMEM) supplemented with 5% fetal calf serum (Biochrom AG, Berlin), penicillin and streptomycin. The cells were incubated in a humidified atmosphere containing 5% CO_2_ at 37°C and passaged every 2–3 days.

### Ciliary activity assay

PCLS were analyzed under a light microscope to estimate the ciliary activity. Each bronchus was virtually divided into ten segments each of which was monitored for the presence or absence of ciliary activity. Slices were selected that showed 100% ciliary activity at the beginning of the experiment.

### Virus propagation

Swine influenza virus of the H3N2 subtype (A/sw/Bissendorf/IDT1864/2003) was provided by Ralf Dürrwald, IDT Biologika GmbH, Dessau-Rosslau, Germany. Virus stocks were propagated in MDCK cells in infection medium (Eagle's minimal essential medium (EMEM)) containing acetylated trypsin 1 µg/ml (Sigma-Aldrich, Munich). Supernatants were clarified by low-speed centrifugation (200×g, 10 min) and stored at −80°C.

Two avian influenza virus strains were used: A/chicken/Saudi Arabia/CP7/98 LPAI of the H9N2 subtype was provided by Hans-Christian Philipp (Lohmann Tierzucht, Cuxhaven, Germany) and strain A/duck/Potsdam/15/80 LPAI of the H7N7 subtype was provided by Friedrich-Loeffler-Institut (Insel Riems, Germany). For propagation, 10-days old specific pathogen-free embryonated chicken eggs (VALO Biomedia, Cuxhaven, Germany) were inoculated with 100 µl of virus solution (virus stock 1∶100 in PBS) into the allantoic cavity of the egg. The eggs were kept at 37°C for up to three days in an egg incubator. Chorioallantoic fluid was collected and centrifuged by low-speed centrifugation (450×g, 15 min) to remove cell debris. Virus stocks were stored at −80°C.

### Plaque assay

MDCK cells were grown in Eagle's minimal essential medium (EMEM) containing 5% fetal calf serum on a 6 well plate for one day. The cells were washed twice by PBS, and infected with serially diluted viral suspensions in EMEM with acetylated trypsin (1 µg/ml). For virus adsorption, cells were kept on a shaker in 5% CO_2_ at 37°C. After one hour, the overlay medium containing avicel microcrystalline cellulose RC 581 (2.5%; FMC Biopolymer, Brussels), EMEM with glutamine (GIBCO BRL Life technologies) and bovine serum albumin fraction V (0.2%; AppliChem) was added in a volume of 3 ml. After incubation for 2–3 days in 5% CO_2_ at 37°C, the cells were fixed and stained with a formaldehyde solution containing 1% crystal violet. The titer of the virus was expressed in plaque-forming units per ml (PFU/ml).

### Virus infection

PCLS were washed twice with PBS and infected with 500 µl of the viral dilution in RPMI medium. For monitoring the course of infection (virus titration, measurement of ciliary activity), virus was applied at a concentration of 10^4^ pfu/ml; slices destined for immunostaining were infected with 10^6^ pfu/ml. Inoculums were removed 2 h.p.i. and PCLS were washed 3 times with PBS before 1 ml of RPMI medium was added as final volume. The slices were incubated for up to 7 days in 5% CO_2_ at 37°C. All experiments were performed at least four times. For each time point, six slices were infected and the supernatants were pooled to determine the infectivity.

### Cryosections

PCLS were mounted on small filter paper with tissue-freezing medium (Jung, Heidelberg, Germany), frozen in liquid nitrogen and kept in −80°C prior to cutting. Slices were cut at 10 µm thickness by a cryostat (Reichert-Jung, Nußloch, Germany). The sections were dried overnight at room temperature and kept frozen at −20°C until staining.

### Immunofluorescence analysis of cryosections

The sections were fixed with 3% paraformaldehyde for 20 min and were permeabilized with 0.2% Triton X-100 for 5 min followed by 3 washing steps with PBS. All antibodies were diluted in 1% bovine serum albumin and incubated with the sections for 1 h at room temperature in a humid incubation chamber. After the last incubation step, the sections were washed three times with PBS and once with distilled water. The slices were embedded in Mowiol and stored at 4°C until examination under the confocal microscope.

For detection of infected cells, a monoclonal antibody against the influenza A virus nucleoprotein (NP) (AbDSeroTec, Düsseldorf) at a 1∶750 dilution was used followed by incubation with an anti mouse IgG (Sigma-Aldrich) secondary antibody.

To detect α2,6-linked sialic acids, FITC labeled *Sambucus nigra* agglutinin (SNA) (Vector laboratories, Burlungame, USA) was used and biotinylated *Maackia amurensis* lectin II (MAAII) was used to determine α2,3-linked sialic acids after preincubation of sections with the Avidin/Biotin Blocking kit (both from Vector Laboratories, USA). The binding of biotinylated antibodies was visualized by incubation of the samples with streptavidin-Cy3 or streptavidin-FITC (Sigma-Aldrich).

Ciliated and mucus-producing cells are commonly stained by antibodies directed against ß-tubulin and mucin, respectively [6], [7], [10], [11]. To visualize cilia, cells were treated with a Cy3-labeled monoclonal antibody recognizing β-tubulin (Sigma-Aldrich). Goblet cells were stained indirectly by using the mucin-5AC antibody (Santa Cruz Biotechnology), followed by an anti rabbit IgG secondary antibody (Sigma-Aldrich). Nuclei of cells were stained by incubation with DAPI (4′, 6′-diamidino-2-phenylindole) for 15 min (37°C).

### Neuraminidase treatment

Mucus was washed away from PCLS were washed with RPMI medium 3 times to remove mucus. Washed PCLS were treated with neuraminidase (NA) type V from *Clostridium perfringens* (Sigma/Aldrich, Munich) at 50 mU/ring and incubated for 2 h at 37°C. After removal of the NA solution, PCLS were washed three times with PBS and then embedded with tissue-freezing medium (Jung, Heidelberg, Germany) to generate cryosections and perform lectin staining.

### Glycan array analysis

#### Preparation of viruses

Allantoic fluid containing influenza viruses was treated with beta-propiolactone at a final concentration of 0.05% (v/v) and incubated for 2 days at 4°C, and sedimented by centrifugation at 60,000 g. The pellet was resuspended in 0.01 M PBS containing 0.05% sodium azide and stored at 4°C. Unless otherwise stated, reagents were obtained from commercial suppliers and used without purification. All aqueous solutions were prepared from destilled deionized water filtered with a Milli-Q purification system and sterile-filtered through a 0.2 µm filter. Anti-H9N2 rabbit polyclonal and mouse anti-influenza A H7 monoclonal antibody were purchased from eEnzyme Inc. (Montgomery village, MD). DyLight 649 goat anti-rabbit IgG antibody and DyLight 649 goat anti-mouse IgG antibody were purchased from Jackson ImmunoResearch (West Grove, PA). Streptavidin-Cy5 was purchased from Invitrogen Corporation (Carlsbad, CA). NHS-coated glass slides (Nexterion - slide H) was purchased from SCHOTT North America, Inc. (San Jose, CA). SuperBlock blocking buffer in PBS was purchased from Thermo Scientific (Rockford, IL).

#### Glycan Microarray Fabrication

A glycan array containing 29 structurally diverse glycans was constructed according to the previous one-pot programmable protocol [25]. Microarrays were prepared by printing (AD3200, BioDot) the glycan with an amide tail to the NHS-activated glass slide (Nexterion H) by robotic pin (SMP2B, TeleChem International Inc.). Nexterion H slides were spotted with solutions of sugar **1**–**29** at 100 µM with 3 replicates for each sugar and then dried under vacuum. The spotted slides were blocked with SuperBlock blocking buffer for 1 h just before use followed with washes by successive rinses in 0.05% Tween in PBS buffer (pH 7.4) (PBST), PBS buffer (pH 7.4), and water three times.

#### Virus Binding Assay

All experiments were repeated three times. Suspensions of the inactivated viruses were overlaid onto the arrays and incubated at room temperature for 1 hour. Slides were subsequently washed by successive rinses in PBS-0.05% Tween, PBS, and deionized water three times. Bound viruses were labeled by primary antibodies. The slides were gently rocked at room temperature for another 1 hour. After the repeating washing steps, binding was detected by overlay with dye-labeled secondary antibodies. The slides were air-dried and scanned with a microarray fluorescence chip reader (GenePix 4300B, Molecular Devices). The PMT gain was set to 450. The resulting images were analyzed with GenePix Pro 7 (Molecular Devices) to locate and quantify the fluorescence intensity of all of the spots on the grid.
